# Innovations in colorectal cancer treatment: trifluridine and tipiracil with bevacizumab for improved outcomes – a review

**DOI:** 10.3389/fonc.2024.1296765

**Published:** 2024-07-12

**Authors:** Taruba Rais, Rumaisa Riaz, Tasmiyah Siddiqui, Amna Shakeel, Afsheen Khan, Habiba Zafar

**Affiliations:** ^1^ Internal Medicine, Dow University of Health Sciences (DUHS), Karachi, Pakistan; ^2^ Internal Medicine, Jinnah Sindh Medical University (JSMU), Karachi, Pakistan

**Keywords:** humans, trifluridine tipiracil drug combination, bevacizumab, United States Food and Drug Administration, prevalence, colorectal neoplasms

## Abstract

Colorectal cancer ranks second in cancer-related deaths throughout the world. At the time of diagnosis, at least 20% of the patients with CRC had already developed metastases. Treating and effectively managing metastatic colorectal cancer remains an unsolved task for the health sector. Research and clinical trials have been done to find the best possible solution for patients diagnosed with metastatic colorectal cancer. The approval of the combination therapy of trifluridine and tipiracil with bevacizumab for previously treated metastatic colorectal cancer (CRC) by the Food and Drug Administration (FDA) is a remarkable breakthrough in CRC treatment. Our goal through this article is to give detailed knowledge about the pathogenesis of CRC, its prevalence, and its clinical features. Here, we have also discussed the past medical treatments that have been used for treating mCRC, including the anti-EGFR therapy, aflibercept, ramucirumab, and regorafenib. However, the focus of this document is to assess the combination of LONSURF (trifluridine/tipiracil) and bevacizumab by reviewing the clinical trials and relevant research.

## Introduction

Colorectal cancer (CRC) occurs when the cells in the colon or rectum’s lining grow uncontrollably, forming a tumor. In terms of cancer prevalence, colorectal cancer is the third most common cancer and it’s the second leading cause of cancer-related deaths worldwide (1). Approximately 20% of CRC patients already have metastases at the time of diagnosis, and half of the patients with localized illness eventually develop metastases (2). This condition requires multiple treatment lines and is often challenging to treat due to the metastatic nature of the cancer. Patients with metastatic CRC are generally believed to have a poor prognosis, with a relative 5-year survival rate of 14%, compared to 90% and 71% in those with localized and regional CRC respectively (3). The evolution of more effective therapeutic techniques, including liver and lung metastases surgery, and new anticancer drugs, has resulted in a prominent improvement in the prognosis for metastatic CRC (mCRC) patients over the last 20 years (4). Despite that, it remains incurable in most cases. New research suggests the combination of trifluridine and tipiracil with bevacizumab as a promising alternative for patients who have previously been treated for mCRC. The Food and Drug Administration (FDA) has approved this combination therapy because of its promising results (5). Numerous clinical trials have evaluated both the efficiency and safety of this combination therapy. In this review, we discuss the pathogenesis and clinical features of metastatic colorectal cancer. Furthermore, this review aims to provide a comprehensive overview of the efficacy and safety profile of this combination therapy in the management of metastatic colorectal cancer. Our goal is to contribute to the current understanding of this treatment option, ultimately improving patient care and outcomes.

## Metastatic colorectal cancer

Colorectal cancer (CRC) ranks as the third most diagnosed cancer globally. Additionally, it is the primary cause of cancer-related deaths in both men and women, following lung cancer. Despite extensive research, the exact causes of colorectal carcinoma remain unclear. Early detection of CRC plays a crucial role in improving patient outcomes; however, individuals with colorectal cancer frequently present with atypical clinical symptoms or only vague signs during the initial stages of the disease, resulting in a low early diagnosis rate (6, 7).

### Epidemiology

Colorectal cancer (CRC) shows geographical variation with more cases reported in developed nations compared to less developed ones. In 2023, the estimated number of adults in the United States who will be diagnosed with colorectal cancer is 153,020 (8). Worldwide, colorectal cancer is the second most lethal cancer for both men and women, following lung cancer. Data from Globocan 2020 reveals that CRC caused 935,173 deaths, accounting for 9.4% of all cancer-related fatalities (9).

### Etiology

The exact cause of colorectal cancer (CRC) remains elusive, but several contributing factors have been identified. Approximately 20% of CRC cases are associated with genetic factors with first-generation relatives of CRC patients facing a three-fold higher risk of developing cancer. Genetic syndromes like Familial Adenomatous Polyposis (FAP) and Lynch syndrome have been linked to hereditary CRC. Non-cancerous conditions like colorectal polyps, adenomas, ulcerative colitis, and Crohn’s disease can contribute to CRC development. Risk factors for CRC have been shown to include age, carcinogenic exposures, smoking, race, gender, IBD, sedentary lifestyle, obesity, and even pelvic radiation therapy (7, 10).

### Symptoms

**Figure 1:**
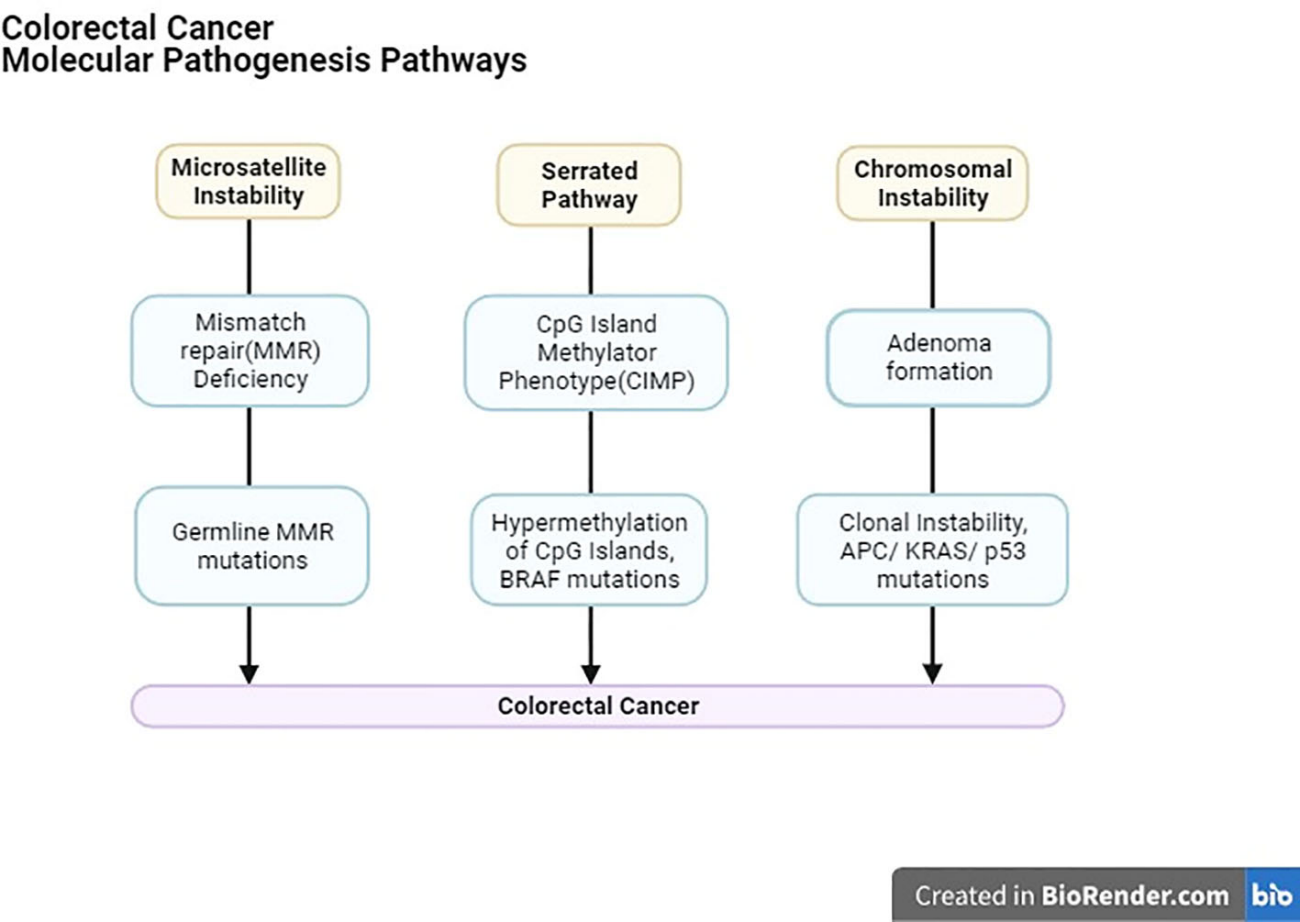
Colorectal cancer: molecular pathogenesis pathways.

Common symptoms that individuals should be aware of include alterations in bowel habits such as diarrhea, constipation, or narrowing of stool. Additionally, symptoms may encompass rectal bleeding which can manifest as either bright red or dark, tar-like stools. Other warning symptoms involve persistent abdominal discomfort, pain, or bloating, unexplained and sudden weight loss, constant fatigue and low energy levels even after adequate rest, and iron deficiency anemia resulting from ongoing bleeding, leading to fatigue, weakness, and pallor (11) (**Figure 1**).

In metastatic colorectal cancer, symptoms can vary based on tumor size and the sites of metastasis. For example, bone involvement may result in pain, fractures, constipation, and elevated calcium levels. Lung metastasis may lead to symptoms like breathlessness, coughing, pain, and fatigue. Liver metastasis can cause nausea, fatigue, swelling, increased abdominal size, and jaundice. Abdominal lymph node involvement may result in bloating, abdominal swelling, and loss of appetite. Additionally, if metastasis occurs in brain or spinal cord, it may manifest as pain, confusion, memory issues, headaches, vision problems, speech difficulties, and seizures (12).

### Pathogenesis

CRC is a multifactorial disease that starts as progressive changes in the surface epithelial cells of colorectal mucosa. Hyperplasia and adenoma formation in these cells can eventually advance into carcinoma. This development is usually initiated by carcinogenic factors which cause alterations in DNA. Morphology includes epithelial hyperplasia, atypical hyperplasia, adenoma formation, carcinoma *in situ*, and invasive carcinoma (13).

**Figure 2:**
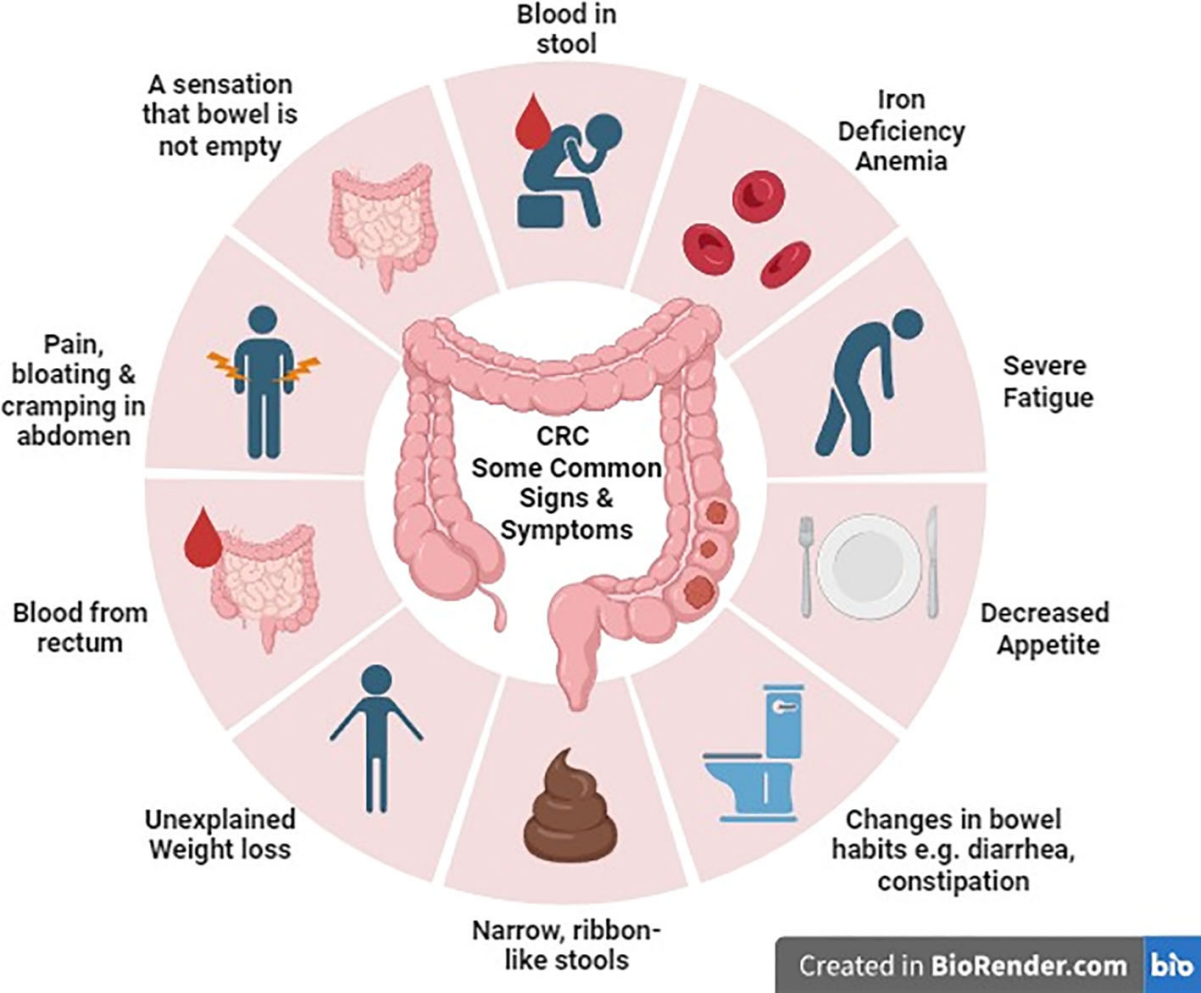
Colorectal cancer: some common signs and symptoms.

With recent innovations in research, notably, three confirmed molecular mechanisms underpin CRC’s occurrence and development (**Figure 2**).

1. Chromosomal instability (CIN), primarily occurring in familial adenomatous polyposis (FAP), involves structural changes in DNA, promoting cancer progression by increasing clonal diversity.
2. Genetic mutations, such as those found in Lynch syndrome and other sporadic mismatch repair (MMR) mutations, play a role in CRC.
3. CpG islands hypermethylation, in specific gene promoter regions contributes to CRC development, sometimes involving the aberration of genes like APC, DCC, P53, K-RAS, c-MYC, MCC, and MMR-related genes.

Importantly, these molecular pathways may coexist in CRC patients (14). Furthermore, genomic and epigenomic instabilities, including chromosomal instability (CIN), microsatellite instability (MSI), non-MSI hypermutability, aberrant DNA methylation, and global DNA hypomethylation, significantly contribute to CRC formation. Understanding these instabilities in CRC, as well as their implications for polyp-to-cancer progression and prevention strategies, is crucial for advancing our knowledge of this disease and improving patient care (15).

Treatment for CRC may vary depending on the stage of the cancer and the extent of its spread. These treatment modalities can include surgical procedures, radiation therapy, chemotherapy, and targeted therapy. The survival rates also vary significantly by stage. Therefore, early intervention is crucial, especially in cases of metastatic CRC (Stage 4) where the five-year relative survival rate is approximately 15.6% (16).

## Past medical treatment

### The anti-EGFR therapy

Cetuximab and panitumumab are monoclonal antibodies developed to specifically address the EGFR (Epidermal Growth Factor Receptor) pathway in individuals with metastatic colorectal cancer, whether they have received prior treatment or are newly diagnosed, even encompassing those with confirmed refractory conditions. Cetuximab is a hybrid antibody combining mouse and human components, while panitumumab is fully humanized. The EGFR protein’s expression levels, assessed through immunohistochemical analysis, are a predictive factor for treatment response. It was assumed that patients with high EGFR expression would benefit more from these antibodies. However, clinical trials in other solid tumors revealed limited predictive value for EGFR expression (17, 18).

Several clinical trials have evaluated the effectiveness of cetuximab and panitumumab in treating metastatic colorectal cancer. RAS and BRAF are crucial genes in the EGFR signaling pathway, serving as biomarkers for mCRC. Mutations in these genes lead to EGFR pathway activation, affecting the efficacy of anti-EGFR targeted therapy (2). The phase III randomized clinical trials demonstrated that individuals with metastatic colorectal cancer and mutations in codon 12 or 13 of the KRAS gene did not experience any advantages from undergoing treatment with these antibodies. The presence of KRAS mutations emerged as a negative predictor, signifying their resistance to anti-EGFR therapy. Patients with KRAS mutations displayed notably shorter periods of progression-free intervals and no discernible survival benefit (19–21).

Similarly, combining these anti-EGFR therapies with XELOX-based chemotherapy regimens did not consistently show benefits (22). In this context, only a slight advantage in response rate was noted in individuals with wild-type KRAS tumors without any noteworthy enhancements in the overall survival rate. Furthermore, studies combining anti-EGFR and anti-VEGF therapies alongside chemotherapy did not yield positive results (23).

### Aflibercept

Aflibercept, or vascular endothelial growth factor TRAP (VEGF TRAP), is a glycoprotein used as a secondary treatment option for metastatic colorectal cancer when the disease progresses after a prior oxaliplatin-based treatment regimen. Its mechanism of action involves binding VEGF-A more strongly than the natural receptors, thus blocking the binding of endogenous ligands to their respective receptors. This action inhibits VEGF receptor activation and endothelial cell proliferation, ultimately preventing the growth of new vessels that supply tumors with oxygen and essential nutrients. Aflibercept binds to PlGF (placental growth factor), acting as a decoy receptor and sequestering PlGF. This prevents PlGF from exerting its angiogenic effects and further contributes to inhibiting new blood vessel formation. In a Velour clinical trial, Aflibercept’s efficacy and safety were assessed in metastatic colorectal cancer patients treated with Oxaliplatin or Avastin. The results showed a significant rise in survival rate, from 12 months to 13.5 months. Additionally, there was a reduction in the relative risk of death and disease progression by 24%. However, despite these promising results, resistance to Aflibercept treatment develops, thus decreasing its benefits. Therefore, negative aspects should be carefully evaluated in the overall treatment plan (24, 25).

### Ramucirumab

Ramucirumab is classified as an IgG1 humanized monoclonal antibody and serves as a secondary therapeutic option for individuals diagnosed with metastatic colorectal carcinoma who experienced disease progression after receiving standard treatment. Its target site is the Vascular Endothelial Growth Factor Receptor-2, making it a unique inhibitor of angiogenesis when compared to other well-known anti-vascular agents like bevacizumab and Aflibercept. Clinical data indicates that when combined with FOLFIRI, Ramucirumab provides a significant advantage concerning the duration of overall survival rate, with an extension of six weeks compared to the standard FOLFIRI treatment alone. However, it’s worth noting that the treatment with FOLFIRI/Ramucirumab did come with some adverse events. The most common ones include fatigue, neutropenia, hypertension, and thrombocytopenia. (26, 27).

### Regorafenib

Regorafenib is a potent inhibitor of multi-kinase that regulates the growth of cancer cells by an intricate system of growth factors and receptors. Regorafenib is a medication used in the treatment of progressive metastatic colorectal cancer (mCRC). To enhance anticancer therapy’s effectiveness, blocking multiple pathways involved in this process is crucial. These pathways include FGFR (Fibroblast Growth Factor Receptor), which is essential for cell differentiation and proliferation; PDGFR (Platelet Derived Growth Factor Receptor), which plays a role in recruiting and maturing pericytes and the TIE-2 receptor, a tyrosine kinase enzyme that plays a role in the formation of new blood vessels (angiogenesis), is present on endothelial cells and is crucial for the development of blood vessels. In human tumors, substances that bind to TIE-2 receptors are frequently increased. Hence, multi-target inhibitors have emerged as a promising research area in cancer treatment. In a Phase III CORRECT trial, regorafenib was evaluated, evaluating metastatic colorectal cancer patients with progressive disease after the initial treatment regimen. Patients were randomized to receive regorafenib and placebo in a 2:1 ratio. Despite the initial promise, it’s important to note that Regorafenib did not meet its primary objectives in phase III clinical trials. While targeting multiple pathways may seem logical, it doesn’t always translate into improved overall survival (OS) rates for mCRC patients. Regorafenib has received authorization for use as a third-line treatment option for patients with limited alternatives, particularly those pre-treated with specific chemotherapy regimens. However, its efficacy varies depending on individual patient factors, including specific genetic mutations (28, 29).

## Recent medication

The Food and Drug Administration has approved the use of LONSURF (trifluridine and tipiracil) along with bevacizumab for adults with metastatic colorectal cancer (mCRC) who have previously received chemotherapy with fluoropyrimidine, oxaliplatin, and irinotecan, as well as anti-VEGF biological therapy, and, if their RAS status is wild-type, anti-EGFR therapy. It’s worth noting that the FDA had previously approved LONSURF as a standalone treatment for this indication back in September 2015 (30).

According to the results of the Phase 3 SUNLIGHT trial, using LONSURF along with bevacizumab has shown significant improvements in overall survival (OS) and progression-free survival (PFS) for patients with metastatic colorectal cancer (mCRC) who have experienced disease progression or cannot tolerate two previous chemotherapy treatments, in comparison to using LONSURF as a standalone treatment. These positive outcomes have resulted in the US Food and Drug Administration designating this combined treatment as a Breakthrough Therapy in August 2023 (31).

### LONSURF^®^ (trifluridine/tipiracil)

LONSURF consists of trifluridine (FTD), an analog of thymidine nucleoside, and tipiracil (TPI), an inhibitor of thymidine phosphorylase, in a precise molar ratio of 1:0.5 (or a weight ratio of 1:0.471) (32). Trifluridine undergoes a series of phosphorylation steps inside cells and subsequently integrates into DNA causing structural damage and dysfunction in the DNA. This is considered the main way in which trifluridine exerts its anticancer effects although it may also play a role in inhibiting the enzyme thymidylate synthase via trifluridine monophosphate (33).

After being taken orally, trifluridine gets rapidly deactivated by thymidine phosphorylase in the intestines and liver, resulting in low oral bioavailability. Nevertheless, when tipiracil hydrochloride, an orally active inhibitor of thymidine phosphorylase, is administered alongside trifluridine, it reduces the initial degradation of trifluridine during its initial journey through the body, increasing its systemic exposure (33).

Based on the findings of a randomized phase 2 study (J003–10040030), TAS-102 obtained approval in Japan in the year 2014 (34). Subsequently, it gained approval from the US Food and Drug Administration in 2015 and the European Medicines Agency in 2016, supported by data derived from the international phase 3 RECOURSE study (35).

During the placebo-controlled RECOURSE trial in Phase III, patients were prescribed FTD/TPI at a starting dosage of 35 mg/m². They took this orally twice a day for five days (Day 1–5) and again for five more days (Day 8–12) within a 28-day treatment cycle. The study indicates that FTD/TPI treatment significantly improved median overall survival (OS) compared to the placebo group (7.1 months versus 5.3 months; hazard ratio [HR]: 0.68). This positive outcome was consistent across various subgroups. Most of the patients receiving FTD/TPI experienced adverse events (AEs) of grade ≥3 severity. However, less than 10% of them reported no non-hematologic toxicities of grade ≥3. Patients reported grade ≥3 neutropenia, anemia, and thrombocytopenia in 38%, 18%, and 5% of cases, respectively, and only 4% of them experienced febrile neutropenia (35). Although it demonstrates effectiveness in various patient subcategories and exhibits a well-tolerated safety profile, trifluridine-tipiracil monotherapy offers only modest improvements in overall survival (36, 37).

LONSURF is authorized for use in patients who are adults and have been diagnosed with metastatic gastric or gastroesophageal junction adenocarcinoma. These patients must have been previously treated with at least two lines of chemotherapy, which included fluoropyrimidine, a platinum agent, and either a taxane or irinotecan. Additionally, if applicable, their treatment must have also included HER2/neu-targeted therapy (32).

### Bevacizumab

Bevacizumab is a well-established cancer-fighting medication that focuses on VEGF, which results in the inhibition of angiogenesis. When used for solid tumors, it is thought to “normalize” the development of abnormal blood vessels within tumors and enhance the distribution of chemotherapy drugs to cancerous tissues (38). This theory is supported by animal studies involving colorectal cancer xenografts, which have demonstrated that when trifluridine is given alongside bevacizumab, it leads to increased levels of phosphorylated trifluridine within cells (39). Furthermore, the inclusion of nintedanib, an angiokinase inhibitor improves the integration of trifluridine into DNA (40).

Bevacizumab (Avastin), marketed as an anticancer drug, received its initial FDA approval in 2004. This approval authorized its utilization as a viable treatment alternative for patients with metastatic colon or rectal carcinoma in the first or second line of therapy, alongside intravenous 5-fluorouracil-based chemotherapy (41). Tebutt’s study found that when directly compared to capecitabine, treatment with Bevacizumab showed a longer Progression-Free Survival (PFS) duration of 8.5 months compared to 5.7 months. However, there were no significant differences observed in Overall Survival (OS), which remained at 18.9 months for both groups (42). The Hurwitz trial aimed to confirm the previous findings by comparing IFL to IFL with Bevacizumab (BEVA). The trial concluded that combination therapy was superior in different aspects, including a higher overall response rate of 45% (compared to 35%), an extended Progression-Free Survival (PFS) duration of 10.6 months (compared to 6.2 months), and a longer Overall Survival (OS) duration of 20.3 months (compared to 15.6 months) (43). The approval from FDA for the use of BEVA in this treatment context was granted due to the compelling results observed in the conducted studies.

## Groundbreaking FDA approval: LONSURF^®^ and bevacizumab combo for metastatic colorectal cancer in adults

“Trifluridine and tipiracil (marketed by Taiho Oncology, Inc. as LONSURF) in combination with bevacizumab received FDA approval on August 2, 2023, for treating metastatic colorectal cancer (mCRC) that was previously managed with fluoropyrimidine-, oxaliplatin-, and irinotecan-based chemotherapy, as well as anti-VEGF biological therapy. It is also approved for mCRC patients with RAS wild-type who received anti-EGFR therapy” (30).

LONSURF, a combination therapy administered orally to address metastatic colorectal cancer, operates through the collaborative influence of trifluridine (FTD) and tipiracil (TPI). FTD, an analog with cytotoxic properties, exerts its anti-cancer effects by integrating into DNA and inducing structural damage. However, its effectiveness is hindered by rapid enzymatic degradation facilitated by thymidine phosphorylase in the intestines and liver. Incorporating TPI, a thymidine phosphorylase inhibitor, mitigates this degradation, resulting in an augmented systemic exposure to FTD. Furthermore, the continuous inhibition of angiogenesis emerges as a pivotal strategy in managing metastatic colorectal cancer. Bevacizumab, renowned for its ability to target vascular endothelial growth factor (VEGF), assumes a critical role in this context by impeding angiogenesis. Demonstrating clinical activity even beyond disease progression, the persistent inhibition of VEGF with bevacizumab proves effective in patients with metastatic colorectal cancer. Through the integration of FTD–TPI with bevacizumab, this therapeutic approach not only harnesses the cytotoxic potential of FTD but also prolongs the suppression of angiogenesis, presenting a synergistic strategy for treating metastatic colorectal cancer. This dual intervention comprehensively addresses the direct impact on cancer cells and the supportive microenvironment, culminating in a comprehensive and productive therapeutic outcome (31).

The pairing of FTD/TPI and bevacizumab has demonstrated effectiveness in treating colorectal cancer (CRC) in mouse xenograft models. Moreover, it has exhibited promising response in the management of advanced mCRC through two initial clinical trials initiated by investigators (39, 44, 45). In both clinical trials (44, 46), the dose assigned and treatment schedule of FTD/TPI mirrored that of the RECOURSE trial (35), Within each 28-day treatment cycle, bevacizumab was given intravenously at a dose of 5 mg/kg on days 1 and 15.

The initial trial, known as C-TASK FORCE (44), was done in Japan in 2014. This single-arm study involved 25 patients with metastatic CRC who had become resistant to all standard therapies. After 16 weeks, the progression-free survival (PFS) rate stood at 42.9% (with an 80% confidence interval between 27.8% and 59.0%). The median PFS was 3.7 months (with a 95% confidence interval ranging from 2.0 to 5.4 months), while the median overall survival (OS) was 11.4 months (with a 95% confidence interval between 7.6 and 13.9 months). Notably, grade ≥3 neutropenia was observed in 18 patients (72%), although the occurrence of febrile neutropenia was relatively low, affecting only 4 patients (16%). Importantly, no adverse events associated with the medication were observed leading to treatment discontinuation (44).

Another trial, conducted in Denmark from 2017 to 2018, 93 patients with refractory metastatic CRC were randomly assigned in a 1:1 ratio to receive either FTD/TPI combined with bevacizumab or FTD/TPI alone (46). In a survival analysis conducted with data up to February 2019, it was observed that FTD/TPI in combination with bevacizumab led to significantly prolonged median progression-free survival (PFS) and overall survival (OS) when compared to FTD/TPI monotherapy. Specifically, the results showed a difference in PFS (4.6 vs. 2.6 months; p = 0.0015) and OS (9.4 vs. 6.7 months; p = 0.028) in favor of the FTD/TPI plus bevacizumab group. Importantly, this disparity in OS persisted even after an additional year of follow-up (9.9 vs. 6.0 months; p = 0.03) (47). Although grade ≥3 neutropenia occurred more frequently among patients receiving FTD/TPI plus bevacizumab, it is noteworthy that treatment-related serious adverse events were infrequent in both treatment groups (47).

The safety and effectiveness of LONSURF with bevacizumab were assessed in the SUNLIGHT trial (NCT04737187), a global study involving 492 individuals with metastatic colorectal cancer (mCRC). These patients had received a maximum of two prior chemotherapy regimens and either experienced disease progression or had intolerance to their last treatment. The main goals of the study revolved around assessing overall survival (OS) and progression-free survival (PFS) as key outcome measures. Statistically significant improvement was observed in OS among patients who were randomized to receive LONSURF plus bevacizumab compared to those who received LONSURF alone (Hazard ratio 0.61; 95% CI: 0.49, 0.77; one-sided p<0.001). Median OS was 10.8 months in the LONSURF plus bevacizumab group (95% CI: 9.4, 11.8) and 7.5 months in the LONSURF group (95% CI: 6.3, 8.6). Median PFS was 5.6 months in the LONSURF plus bevacizumab group (95% CI: 4.5, 5.9) and 2.4 months in the LONSURF group (95% CI: 2.1, 3.2) (Hazard ratio 0.44; 95% CI: 0.36, 0.54; one-sided p<0.001) (31). The improvements in OS and PFS observed with the combination of LONSURF and bevacizumab were linked to the preservation of the patients’ quality-of-life from the beginning of the treatment until cycle 6. Notably, there were no noteworthy alterations in mean scores within any of the subdomains assessed in the EORTC QLQ-C30 and EuroQol EQ-5D-5L health-related quality-of-life questionnaires (45). The frequently observed adverse events and laboratory abnormalities in LONSURF plus bevacizumab-treated patients (occurring in ≥20% of cases) included neutropenia, anemia, thrombocytopenia, fatigue, nausea, increased AST, increased ALT, increased alkaline phosphatase, low sodium, diarrhea, abdominal pain, and decreased appetite (31).

This coupling of LONSURF with bevacizumab did not elevate the risk of serious adverse events or events leading to treatment discontinuation. When FTD–TPI was combined with bevacizumab, superior survival outcomes were observed compared to FTD–TPI alone, particularly in most prespecified subgroups of patients. The safety profile of FTD–TPI plus bevacizumab aligned with expectations, and individuals who received the combination exhibited a prolonged preservation of performance status compared to those treated solely with FTD–TPI. This compelling body of evidence strongly supports the rationale for adopting the trifluridine/tipiracil and bevacizumab combination as a promising and well-tolerated therapeutic approach in the management of metastatic colorectal cancer.

## Clinical trials showing effects of bevacizumab plus trifluridine/tipiracil on pre-treated colorectal cancer

Evidence from multiple clinical trials and preclinical studies has demonstrated the combination of trifluridine and tipiracil along with bevacizumab as a remarkable treatment regimen for heavily pretreated metastatic colorectal cancer (mCRC). According to a phase 2 trial carried out by Pfeiffer et al. that recruited around 93 patients, TAS-102 with bevacizumab demonstrated clinically notable enhancement in median progression-free survival from 2.6 months due to TAS-102 monotherapy to 4.6 months in the management of metastatic colorectal cancer that was unmanageable through chemotherapy along with exceptionally well-tolerated safety profile. In terms of safety, the combination group was comparatively safer than the monotherapy except for the severe neutropenia which was higher in the combination group. Since the efficacy of trifluridine and tipiracil has been shown by numerous trials, the aim of this study was to demonstrate its effect along with bevacizumab, however, small number of randomised patients together with assessment of PFS by investigator and lack of independent committee’s validation could limit the reliability of results (47).

Considering the analysis of different studies, a retrospective observational analysis, 36 patients insensitive to mainstay therapy were given TAS-102 2 times a day for days one to five and eight to twelve for 4 weeks with Bevacizumab on the first and fifteenth day. Many patients received this treatment as third-line therapy. After an average follow-up of 11.6 months, the median progression-free survival was 4.3 months, and the median OS was 9.3 months. The therapy showed adverse effects of Neutropenia (74.3%), asthenia (65.7%), anemia (54.8%) and thrombocytopenia (34.3%) with no reports of febrile neutropenia and deaths related to the treatment. This study reveals the efficacy of TAS-102 with bevacizumab in subjects with refractory CRC in regular clinical use (48).

Enforcing the results of the aforementioned trial, FTD/TPI and bevacizumab combination in a global phase 3 SUNLIGHT trial that included a total of 246 patients, notably extended the PFS from 2.4 months to 5.6 months. Moreover, it showed significant prolongation of overall survival from 7.5 months with a 30% rate at 12 months without bevacizumab to 10.8 months with a 43% rate with bevacizumab. It is particularly noteworthy because this is the only trial that has exhibited the clinically significant efficacy of bevacizumab after second line therapy as a third line therapy. Additionally, it exceptionally made the effects of this combination evident in patients in which RAS mutation was carried by around 69% of subjects, a higher than commonly recorded percentage of this type of mutation in contrast to other trials which established only marginal overall survival benefits in RAS mutated CRC patients. Safety outcomes in both groups were comparable, however, as observed previously, severe neutropenia was reported to be higher in the combination group than in the monotherapy group (49). These supreme changes in survival rate provoke the accelerated endorsement of this combination by the Food and Drug Administration (FDA) (50).

In order to investigate the efficacy and safety of Trifluridine/Tipiracil plus bevacizumab in patients with heavily pretreated mCRC, a retrospective cohort study was conducted in which 66 patients enrolled with CRC received Trifluridine/Tipiracil with Bevacizumab therapy. The study showed significant increase in the Median progression-free survival as 3.7 months with the combination therapy while it was reported to be 2.2 months in patients receiving the monotherapy and the PFS rate at 16 weeks was reported to be 46.6% and 33.9% respectively. The study reveals that patients in the Trifluridine/Tipiracil plus bevacizumab had significantly longer PFS and median OS. For safety, the therapy reported a relatively greater prevalence of grade >3 Neutropenia in people with pretreated CRC receiving the combination therapy than in the Trifluridine/Tipiracil monotherapy group as observed previously. Therefore, this study reveals that trifluridine/tipiracil plus bevacizumab helps prolong PFS in patients with previously treated mCRC exhibiting tolerable adverse effect (51).

To assess the safety of Trifluridine/Tipiracil with bevacizumab as a combination therapy for patients with metastatic colorectal cancer, a the phase II study with 19 patients receiving the combination treatment of Trifluridine/Tipiracil with bevacizumab was conducted. All the patients received the combination therapy biweekly as third-line chemotherapy. The study indicated the median progression-free survival was 5.6 months and OS was 11.5 months. Five patients received response and the control rate of disease came out to be 9 out of 12. Only a single patient encountered treatment-induced side effects of neutropenia (grade 3 or above) which consequently highlights the safety of the therapy. Hence, this study reveals that the combination of TAS-102 with bevacizumab accelerates tumor depreciation by decreasing the prevalence of neutropenia, improving survival, and represents an effective therapeutic choice for advanced-stage mCRC patients undergoing third-line treatment or beyond (52).

On top of its extraordinary effect on survival, combination of TAS-102 with bevacizumab possesses exceptional antitumor activity than either agent alone as proved by a study published in Oncology reports where this composition exhibited better results on relative cancer volume following fourteen days after drug intake and period consumed for the relative cancer volume to grow five times (53).

To evaluate whether the efficacy of TAS-102 could be improved by combining it with Bevacizumab, in a retrospective study, a total of 57 patients were observed. 21 patients were administered TAS-102 with bevacizumab and 36 patients administered TAS-102 without bevacizumab were enrolled. The median OS was recorded at 14.4 months in the T-B category (patients receiving TAS-120 plus bevacizumab) and 4.5 months in the T group (patients receiving TAS-102 monotherapy). The median TTF was also significantly longer in T-B group (5.6 months) than in T group (2.1 months). Comparing the efficacy and safety, the study recorded a greater incidence of hypertension in the T-B category than in the T category (23.8% vs. 0.0%, p = 0.005). The polytherapy of TAS-102 with bevacizumab significantly enhanced the overall survival, high antitumor activity and other indicators, associated with improved clinical outcomes in subjects with mCRC (54).

Consequently, in the phase Ib/II trial evaluating biweekly combination therapy with TAS-102 and BEVA, 46 patients intolerant to chemotherapy were given TAS-102 with bevacizumab. The median progression-free survival was reported to be 4.29 months and overall survival was 10.86 months. The disease control rate is 59.1% with adverse effects such as leukopenia (15.9%), neutropenia (15.9%), and hypertension (40.9%). This study concludes that TAS-102 plus Bevacizumab encourages anti-cancer efficacy with a favorable safety profile (55).

Besides, a pooled analysis of 29 studies conducted by Yoshino et al. displayed its clinical efficacy as DCR associated with the combination was 64% than FTD/TPI alone, similarly, increment in median PFS was seen from 2.6 to 4.2 months, 3% to 9% at 12 months, 8.1 months to 9.8 months of median OS and 38% from 32% at 12 months. However, huge gap between patients in the intervention group (n=289) and in the control group (n=9383), high heterogeneity and publication bias may serve as confounding bias and challenge the authenticity of these results, particularly important effect of heterogeneity on pooled Kaplan- Meier curves may influence the accurate interpretation of the reported results. Safety profiles of both groups were similar except for grade 3 neutropenia which was higher in FTD/TPI + BEVA group than in the monotherapy group, however, these results are not fir for clinical implication owing to inconsistency in the reporting of adverse events across the studies (56). These clinical trials showing the key findings of clinical trials on Trifluridine/Tipiracil plus Bevacizumab are mentioned in **Table 1**.

**Table 1:** Summary of key findings of the included clinical trials on Trifluridine/Tipiracil plus Bevacizumab.

Study	Sample size	Phase of trial	Survival rate (months)		Outcomes reported	Adverse events
			OS	PFS		
Pfeiffer et al	93	Phase 2	-	4.6	Progression Free Survival	Grade>3 febrile neutropenia, fatigue, diarrhea, nausea, vomiting, obstipation
Martínez N et al SUNLIGHT	36 246	Retrospective Study Phase 3	9.3 10.8	4.3 5.6	Progression Free Survival, Overall Survival Progression Free Survival, Overall Survival	Neutropenia, asthenia, thrombocytopenia, anemia, nausea, vomiting Neutropenia, asthenia, thrombocytopenia, anemia, nausea, vomiting, fatigue, diarrhea, decreased appetite, constipation, stomatitis, hypertension, decreased neutrophil count
Kotani D et al	66	Retrospective Cohort	-	3.7	Progression Free Survival	Neutropenia, asthenia, thrombocytopenia, anemia, nausea, vomiting, fatigue, diarrhea, decreased appetite, proteinuria, anorexia, febrile neutropenia, hypertension, leucopenia
Ishizaki T et al	19	Phase 2	5.6	11.7	Progression Free Survival, Overall Survival	Grade 3 or more neutropenia
Fujii H et al	57	Retrospective Cohort	14.4	-	Progression Free Survival	Neutropenia, thrombocytopenia, anemia, nausea, vomiting, fatigue, diarrhea, malaise, proteinuria, anorexia, febrile neutropenia, hypertension
Satake H et al	46	Phase 2	10.8	4.29	Progression Free Survival, Overall Survival	Decreased white blood cells, neutrophils decreased, anemia, platelet count decreased, alanine aminotransferase increased, aspartate aminotransferase increased, blood bilirubin increased, creatinine increased, proteinuria, anorexia, febrile neutropenia, hypertension, nausea, vomiting, fatigue, peripheral sensory neuropathy, mucositis oral, Palmar-plantar erythrodysesthesia syndrome, Weight loss, Dry skin, Alopecia, Dysgeusia, EpistaxisRash acneiform, Thromboembolic event

OS, Overall Survival; PFS, Progression Free Survival.

### Advances in therapeutic strategies for inflammatory bowel disease-associated colorectal cancer: targeted interventions and prognostic insights

Inflammatory bowel disease (IBD) includes ulcerative colitis (UC) and Crohn’s disease (CD). IBD is associated with increased risk of CRC due to chronic inflammation, genetic factors, and disrupted gut microbiota interactions. This elevated CRC risk is driven by inflammation-induced dysplasia, leading to colitis-associated CRC (IBD-CRC) (57). Due to aggressive histological characteristics and reduced resectability of the tumors, the prognosis of IBD-CRC tends to be more unfavorable than sporadic CRC. Chronic inflammation leads to specific molecular mechanisms, which cause oxidative stress-related damage and DNA double-strand breaks. It eventually leads to increasing levels of molecular markers linked to oxidative injury (58). However, the precise pathogenesis of IBD-related CRC remains a subject of ongoing research.

Both sporadic CRC and IBD-CRC share pathogenic features such as chromosomal and microsatellite instabilities, along with DNA hypermethylation. However, unlike sporadic CRC, IBD-CRC typically does not follow the conventional adenoma–carcinoma sequence. Instead, it progresses from low-grade dysplasia (LGD) to high-grade dysplasia (HGD) and eventually to CRC (58). Recent research suggests an alternative “Big Bang” model of CRC development in the context of inflammation. In this model, multiple tumor-initiating mutations arise simultaneously in a single clone rather than gradually accumulating over time. This accelerated mutagenesis is believed to result from colon inflammation, leading to abrupt tumor initiation (59).

Owing to the clear correlation between IBD and CRC, utilization of drugs mainly immunosuppressant and non-steroidal anti-inflammatory drugs could significantly pave the way in reducing the risk of IBD- related CRC. While identifying various prognostic factors for colorectal cancer in patients with IBD, a meta-analysis of 164 studies, conducted by Wijnands AM et al. demonstrated reduced possibility of CRC in patients who received 5- Amino salicylic acid (5- ASA) (60). Enforcing these results, another meta-analysis that included around 15,000 subjects showed 5-ASA to have chemo preventive effect in the development of CRC in IBD patients with optimal maintenance dosage of 1.2g/day or more (61). Not only 5-ASA, thiopurines also play a crucial role in lowering the risk of CRC in IBD patients, as indicated by the decreased beta catenin activity after topical administration of thiopurine thioguanine in a murine model of colitis associated cancer (62). Similarly, another pooled analysis of 11 cohorts and 16 case control studies displayed protective effects of thiopurines against CRC in IBD patients, particularly in those who had prolonged duration of illness (more than 8 years) (63). Anti-tumor necrosis factor alpha agents have also been remarkable in prevention of CRC in IBD patients. A large multicenter study evaluating 62,007,510 patients showed that IBD patients treated with anti- TNF agents had less likelihood to develop CRC. (64) Ongoing research is crucial for understanding IBD-CRC mechanisms, developing new biomarkers, and implementing early detection strategies to mitigate the impact of these cancers on public health.

## Conclusion

The combination therapy of trifluridine and tipiracil with bevacizumab has exhibited promising outcomes in the treatment of previously treated metastatic colorectal cancer. This comprehensive overview has shown the efficacy of combination therapy in improving the health of mCRC patients. In addition to that, therapy with trifluridine and tipiracil along with bevacizumab has a manageable safety profile, with the most usual adverse complications being neutropenia, fatigue, and diarrhea. The FDA has approved this combination therapy as it expands the treatment options for patients with mCRC and supports the overall survival of these individuals. At the same time, it is still important to note that this therapy may not be suitable for every patient, and an individualized treatment plan should be considered for such patients. Continued approval of this therapy depends on the clinical benefits and favourable prognosis, so further research and clinical trials should be done to attain maximum efficacy.

## Author contributions

TR: Conceptualization, Supervision, Writing – original draft. RR: Writing – original draft. TS: Writing – original draft, Writing – review & editing. AS: Writing – original draft. AK: Writing – original draft. HZ: Writing – original draft.
